# "The Hospital" Nursing Mirror

**Published:** 1897-07-17

**Authors:** 


"The Hospital" Nursing Mirror.

Being the Nursing Section of "The Hospital."

[Contributions for this Section of "The Hospital" should be addressed to the Editor, The Hospital, 28 & 29, Southampton Street, Strand, London, W.C., and should have the word "Nursing" plainly written in left-hand top corner of the envelope.]

### News from the Nursing World.

### THE PRINCESS AND THE COACHMAN.

The Princess May's visit to the wards of the National Hospital for the Paralysed and Epileptic gave great pleasure to the patients. She spoke to many, and made special enquiries about the Princess Christian's coachman, who had been admitted the previous day suffering from the after effects of influenza. One of the objects for which the above hospital is raising funds is "improvements in the provision for the nursing staff." This is much needed, especially in the "women's wing," and we trust that the sum required will soon be in hand. The hopelessness which attends many brain afflictions makes it most desirable that the nurses in charge should be well cared for, and we congratulate the authorities that they have considered the subject worthy of a public appeal.

### HOW THE PRINCE AND PRINCESS WERE WELCOMED.

The route from Lewisham to Hither Green traversed by the Royal party was gaily decorated with Venetian masts, garlands, and flags; but the crowded border of faces, young and old, hale and halt, of all kinds and degrees bearing a universal expression of loyal good-will, was the finest adornment of the Royal progress. Before the infirmary were a row of invalids in their movable chairs, and behind them were grouped their cheery-faced nurses, both holiday-making in honour of the event.

### TO HELP THE PLAISTOW MATERNITY CHARITY.

The fourth annual meeting of the supporters of the Maternity Charity and District Home at Plaistow was held at Stafford House, and was presided over by the Duchess of Sutherland. Again the right note was struck when Mr. George Wyndham moved a resolution to the effect that it "was essential to the welfare of the charity that it should be freed from debt, and a more regular income provided to meet current expenses." The way charities entangle themselves in financial difficulties, and trust to the moneyed man to extricate them, has a most demoralising effect. When money fails, curtail the charity, and so bring home to the people the fact that, if they will not bear their share of responsibility, neither shall they reap the benefit. It is the only way to rouse the well-to-do middle class to their duty in this matter.

### HONITON NURSING ASSOCIATION.

This association and the medical profession, as represented by Drs. Macaulay and Shortridge, are in anything but agreement. The facts of the case appear to be these. The manner in which the association was first constituted was not acceptable to the medical men, consequently the committee reorganised it, consulting them on every point; yet they are dissatisfied, so much so that Dr. Shortridge wrote to Mr. Avery, the hon. secretary, saying that, if a nurse were sent to any of his patients, he would retire from the case. This is certainly overbearing, and Mr. Avery was quite in the right when he said that a nurse would be sent to anyone requesting her services. Drs. Macaulay and Shortridge also desire to forbid the services of the district nurse to anyone earning more than £1 a week. We are sorry to see this unfriendly spirit abroad. The medical man is undoubtedly the supreme authority with his patient, but he has no right to interfere with that patient's choice of a nurse, if she be suitable to the case, because he objects to the association that provides her.

### CHELMSFORD'S PROJECT.

At a meeting under the presidency of the Rector of Chelmsford, Dr. J. C. Thresh (county medical officer) formulated a scheme to supply the district with nurses. He suggested the formation of an association, which he thought one-third of the parishes would join. If a rate of 10s. per 100 population were subscribed, about £70 of the £100 needed would be realised. Three nurses would be required, one to reside in Chelmsford. The idea was approved, and a committee appointed to carry out preliminary steps.

### IN SPITE OF PREJUDICE.

Torpoint has a nursing association to commemorate the "Queen's Year." It is to be called the "Antony and St. John's District Nursing Association," and consists of the medical men, clergy, and subscribers. There will be a president, a vice-president, and a committee of twelve ladies. All that is now needed is a nurse with tact, as the scheme has met with considerable opposition through prejudice.

### WORKHOUSE INFIRMARY NURSING ASSOCIATION.

The thirty-eighth quarterly letter to the Mary Adelaide nurses lies before us. Our Queen and the achievements of her reign, especially as regards medicine and nursing, is the subject, and it is handled concisely and inclusively. During 1897, seventeen nurses obtained the "Mary Adelaide" medal, of which number thirteen were trained by the association. The medals were presented on the occasion of the seventeenth annual gathering; and, at the same time, thirty Prince of Wales's Hospital Stamps were given to the nurses.

### GRIMSBY AND DISTRICT NURSES.

The seventh annual report of the Nursing Institution has been received. There are two staffs, one of twelve nurses engaged in private work, and the other of three nurses, who visit the sick poor of the district gratuitously. Two hundred and twenty-eight patients were visited up to the end of last year. New and more commodious premises have been secured by the committee. On the ground floor are two large sitting-rooms, dining-room, kitchen, &c. Each district nurse has a private bed-room, and those on the private staff have curtained-off cubicles. The situation is in the best part of the town, and the directors of the tram company have given the nurses free passes, which they find a great boon.

### SHOULD DISTRICT NURSING BE A CHARITY?

Some medical men, whilst acknowledging the benefit, nay, the crying need for trained district nurses, disapprove of the charitable basis upon which the supply is furnished. They contend that it tends to pauperise the poor, and that, although paupers might obtain the help of the nurse in illness through the instrumentality of the guardians, yet organised subscriptions of a small amount, on the plan of dispensary medical aid, would suffice to maintain an adequate number of nurses. This would undoubtedly be a desirable state of things, and one to which the promoters of nursing charities are keenly alive, but the poor require a considerable amount of education before they will appreciate the boon of good nursing, and some external aid is necessary in order to fight the ignorance and old bad methods before they will willingly tax their resources to provide what to many of them seems an irksome innovation.

### A WELL-EARNED TESTIMONY.

The nursing school attached to the Cama Hospital, Bombay, is doing excellent work since its foundation by means of a fund raised by Lady Reay in 1886, and increased by Lady Harris. Ninety-eight nurses have received certificates. During the plague several pupil nurses volunteered for work at the Arthur Road Hospital, and the Mother Superior of All Saints' Sisterhood, in writing her thanks to their matron, Miss Atkinson, bears this testimony to their efficiency: "The sisters under whom your nurses have worked speak most highly of them. They are most capable and nurselike, and are always ready to do what helps most, and I hardly think that any nurses could have had a more trying time than they had at Arthur Road I am sure that none of us can forget how the nursing staff at the Cama Hospital came to the rescue in time of need." The Mother Superior sent a cheque for Rs. 300 towards the Bombay branch of the Countess of Dufferin's Fund. A band of devoted nurses—Miss Oran, Miss Steel, and Miss Franklin, Miss Goulden, Miss Sykes, Miss Briggs, Miss Mitchell, and two young ladies recently trained in the Parel Government House Plague Hospital, having done excellent work at Bombay, have followed the foe to Cutch Mandoie, and will, we hope, soon drive it from its new stronghold.

### FRAUDULENT DIPLOMAS.

The French Midwives' Association and that of the Departement de l'Oise are endeavouring to obtain redress in a somewhat curious case of fraud. Certain women, without training, or in any way complying with the prescription of the law, obtained diplomas by underhand means, and are now openly practising the calling of midwifery. One even is in the employ of a hospital. Naturally the properly-qualified midwives objected, and petitioned their members of Parliament to get these falsely-obtained diplomas cancelled. The Minister de l'Instruction Publique et de la Justice, before whom the matter came, replied that, although fraud had been practised, and means would be taken to prevent its repetition, still he did not consider it his duty to follow the affair further, and get the falsely-obtained diplomas annulled by the Council of State (Conseil d'Etat). The legal aspect of the affair is droll. The law is broken, but no penalty is exacted; a minister of the law does not consider it his duty to restrain quacks practising with fraudulently-obtained diplomas; and, lastly, an orthodox body of public servants have to go before Parliament to obtain redress in a case with which the ordinary tribunals of the country ought to be fully qualified to deal.

### DISTRICT NURSES FOR ARGYLLSHIRE.

Miss Guthrie Wright, secretary of the Queen's Nurses' Institute, Edinburgh, addressed a meeting at Argyll Lodge of ladies and gentlemen hailing from North Britain, explaining the principles and details of district nursing. After discussion a scheme was formulated, and a provisional committee formed; and Sir Donald Smith announced his intention of giving a yearly subscription of £150 to the County Association.

### A NEW NURSES' HOME.

The new nurses' home of the isolation hospital at Hither Green, S.E., is very large, and affords accommodation for 191 nurses. Each nurse will have—for as yet it is not finished—a bed-room to herself furnished in light wood. The usual hospital beds, with spring and hair mattresses, a corner washstand, with marble top and blue and white ware, a combination wardrobe and dressing chest. There are two dining-rooms, one for the staff nurses and the other for the nurses, two sitting-rooms, one for each, and a writing-room common to both, and one bath to eight nurses. The walls are distempered in different tints, the flooring in the common rooms is inlaid teak, that in the bed-rooms sound-proof asphalte. There are three entrances, one at each end and one in the centre. The home is situated at the further side of the hospital, and that portion of the grounds assigned to the nurses borders on the open country, and has a number of beautiful trees.

### SHOULD UNION NURSES BE WORKHOUSE TRAINED?

Mr. W. Malden, M.A., M.B., medical officer of Tonbridge Workhouse, read an excellent paper at a recent meeting of the Workhouse Masters' Association. He pointed out several anomalies in the present rules, and states that "the general course of failure under the present system is that the nurse's position is not what it ought to be." The master and matron are now, responsible for the "direct care of the sick unless there were separate institutions." A well-trained nurse naturally resents being under untrained supervision. To remedy this defect her office ought to be put on the same footing as that of the schoolmistress, and she should be responsible as regards nursing arrangements to the medical officer, and to the master in things that appertained to domestic matters. He is of opinion that infirmary nurses ought to be infirmary trained, as the fuller, more stirring life of the hospitals makes the more monotonous duties of the infirmary dull by contrast. We need hardly repeat our conviction that the position of the infirmary nursing is in many cases unsatisfactory, and that the sooner it is put on a common sense footing under properly qualified nurses the better for all parties concerned—the sick, the nurse, the master and matron, and the guardians.

### Lectures to Surgical Nurses.

By H. A. Latimer, M.D. (Dunelm), M.R.C.S. of Eng., L.S.A. of London, Consulting Surgeon, Swansea Hospital; President of the Swansea Medical Society; Lecturer and Examiner of the St. John Ambulance Association, &c.

(*Continued from page 57.*)

### VIII.—INFLAMMATION: ITS PHENOMENA AND SYMPTOMS — DEFINITION OF THE TERM "TISSUE."

Having instructed you about wounds and the methods by which Nature repairs them, I will now consider the processes which arise from time to time to complicate their being healed. Foremost amongst these processes is the one known as inflammation—a state which has been well defined by Green as "the succession of changes which take place in a living tissue as the result of some kind of injury, provided that this injury be insufficient immediately to destroy its vitality." As the word "tissue" which occurs above is one which you will frequently meet with, it will be well, I think, to explain its meaning. You will hear the word used frequently in the wards of a hospital, or wherever medical men are engaged in discussing the changes, wrought by disease, in the body. In anatomy it means the texture or organisation of parts, and in this sense it is applied to any structure in the body. Thus—we speak of bony, muscular, nerve, fatty, or other tissues, meaning thereby bone, muscle, nerve, or fat. When, then, any tissue is irritated or injured to a degree not sufficient to kill it there and then, the process known as inflammation sets in; and I want you to fix this fact firmly in your minds with reference to inflammation, that this process is everywhere the same, in whatever part of the body it is taking place. Four symptoms mark it—redness, heat, pain, and swelling—and we will proceed to examine these symptoms, and to account for them on scientific lines, studying them for that purpose in a part which can be seen and which is not rarely affected by irritation and its resulting inflammation. Let us suppose, then, that an irritating particle of dust has alighted upon the surface of the eye. You have all seen what results in such a case. The eye becomes "blood-shot," it smarts, becomes hot, and, if the irritant is not quickly removed and something of a soothing nature is not quickly applied, swelling of the eyelid results. Fix this common, everyday example of inflammation of the eye on your mind, and you will have an ever-present example of the inflammatory state before you. The eye becomes red, or "blood-shot," as it is called, because small blood vessels, which are ordinarily indistinguishable on account of their minute size, become dilated and occupied by a larger amount of that red fluid, the blood, than they held before the irritation had affected the part; it gives a sensation of heat because more blood is contained there. Pain is felt as a result of pressure upon the sensitive nerves of this extra amount of blood, and by the last link in this chain of phenomena which accounts also for the swelling, viz., the fact that, after a time, the fluid of the blood, and many of its cells also, escaped from the vessels and crowded into the surrounding tissues, filling them up, and caused them to become swollen and puffy. I do not propose to weary you with a minute description of the changes which occur in the sizes of the blood vessels, or of the state of the circulation of the fluid within them; it will suffice to say that, with respect to the latter, if the inflammation continues, the blood ceases to flow altogether, and that, owing to an alteration in the life of the vessel walls, fluid and white and red cells of the blood, after adhering to the same for a time, work their way through them and emigrate into the textures around, and so cause changes which are of more or less consequence, and which I shall have to speak of presently when considering the results of inflammation. What I particularly want you to note now, as nurses, is that the pain which accompanies inflammation has distinctly different characters according to the tissue in which the process in question is being carried on, and that you should learn the terms which are applied to the varieties of it, with a view to understanding their signification. Pain, then, is said to be of a stabbing kind when it accompanies pleurisy, pericarditis, and inflammation of other serous membranes; to be aching when bone is affected; to be throbbing when matter has formed, or is forming; and to be of a bursting variety when the process is being carried on in an organ which has a tense or tight sheath, or enclosing bag, as in the eye. Learn to be very particular as to the expressions patients make of their sufferings, for although their descriptions of their sensations are often most fanciful and ridiculous, they convey a very suggestive meaning, and are indicative both of the source of pain and of its mode of origin.

### Home and Infirmary for Sick Children, Lower Sydenham.

In the midst of all the Jubilee festivities everything has been done to give sick children happiness and enjoyment—what our American cousins would call "a good time."

On Tuesday, June 22nd, the day of the Jubilee procession, by the kindness of the ladies who work at the home, every child, both at Sydenham and at the convalescent branch at Broadstairs, was regaled with a sumptuous tea of strawberries and cake. Mrs. Eschwege had kindly presented Jubilee medals to the children, nurses, and household, and these were worn on Jubilee Day. The gift of a doll or other toy to each child completed the happiness of the little ones. The National Anthem appropriately closed the day's enjoyment. The Jubilee treat proposed was, however, deferred to Wednesday, June 30th, which was a day of great interest in the history of the home, being the twenty-fifth anniversary of its opening. It started with one child, and fifty-four was the number present on Wednesday last.

The home and its grounds had been rendered gay by flags, which must have numbered hundreds, and which were lent by kind friends for the occasion. Tea was served on the lawn for all the little patients who could be out, and, thanks to most favourable weather, very few had to stay indoors. Strawberries and other delights, including a huge birthday cake, were thoroughly appreciated by the children, who were waited on by the nurses and many other kind friends. Amongst those present were Miss Elwes, the Misses Attree and friend, Mr. Aste and family, Mr. E. M. Stone, Mr. and Mrs. F. Carter, Drs. Collins, Perkins, Parnell, and Grayling, the Revs. F. H. Francis and R. T. Ellis, and, of course, Miss Meadows, the matron, and Mrs. Gray, who superintends the Broadstairs branch. After the tea the ladies and gentlemen present, with the staff and household, went to the committee room, where a presentation was made to Miss Meadows by Miss Elwes, the president of the home, on behalf of the committee and other friends. Miss Elwes spoke of Miss Meadows' many years of loving work, and of their association in the work of the home from its very beginning. She presented to her on behalf of the subscribers an illuminated address and a purse containing £40. Hearty applause greeted Miss Meadows on accepting the testimonial, and the party then returned to the garden, where a capital "Punch and Judy" show and a distribution of Jubilee mugs gave the finish to a very happy day.

### Post-Graduate Clinics for Nurses.

By a Trained Nurse.

XXII.—NURSING KINDERGARTENS AND MASSAGE.

I have seen much better results accruing from the treatment of a male rest-patient at home than would be possible in the case of a woman, for the man is not haunted by those domestic spectres—the waste in the kitchen and the general collapse of the carefully built-up fabric of household structure —which usually beset the bedside of the average sick woman. The masculine rest-patient is not particularly interested in the domestic cuisine, so long as he gets his meals sent up fairly comfortably, and the crying of the baby, which racks the woman heart, affects him only so far as it is a "noise" to be stopped. So that I must confess to having seen some fairly good samples of men rest-patients treated at home. There are only two factors which militate against a man patient and his complete rest-cure amid domestic surroundings. These two are the wife and the business. "If a man have a sensible wife warranted not to weep over his woes, and if he can be kept from interviewing clerks and business folk, I allow him to remain at home for rest-cure," is a familiar axiom from the lips of a successful nerve doctor. On the other hand, it must be borne in mind that no victim to a "habit" of any kind—from opium down to tea or tobacco—can be treated at home, for it s quite impossible in these cases for a nurse to successfully keep watch and ward over a staff of servants. Hence some smuggling of contraband goods into the sick-room is nearly sure to take place so long as such a patient remains in his own household, where he may exercise his authority as an employer, and suasion in the form of "tips" for forbidden luxuries procured.

Partial rest-cure—that is to say a modified form of the treatment—is sometimes employed in the incipient stages of nervous exhaustion, and may be carried out at home. Many patients who have perhaps had a previous breakdown, and are wise enough in their generation to recognise the value of precautionary measures, appeal to their physician when they first feel the old symptoms of tire, nerves, and want of self-control coming on. In these cases it is not uncommon to put a trained nurse in charge of the patient, in order that a proportion of rest and seclusion may be enforced each day. Such a nurse will see that the patient threatened with nerve prostration adopts just so much of the habits of a semi-invalid as is salutary. She will enforce breakfast in bed, periodic lyings down through the day, will perhaps give massage and the daily sponge bath while the patient lies in bed. It will also be her duty to carefully watch over the diet of her patient, prevent over-stimulation either by tea or alcohol, and generally arrange the best hygienic conditions as to hours of sleep, exercise, ventilation, and rest. It will be part of her province to keep the patient bright and cheerful, to read aloud to her, and altogether to play the part of a pleasant nurse-companion. A nurse in charge of such a case has often a very agreeable and easy position, especially since favourite prescription in some of these instances is that the patient go each Friday to Monday to country or sea. It is found that many women overweighted by family cares, and who allow themselves to take butchers' bills and other housewifely worries too seriously, find great benefit from an occasional partial rest-cure such as this. Indeed, in the United States many busy housekeepers make it a rule to spend every "week-end" at the quiet home of friends or in secluded country lodgings. This partial rest-cure—for that is what it resolves itself into—has a wonderful effect in calming and soothing irritable nerves. Just as the habit, by no means uncommon in England for hard-working persons,—such as hospital matrons and sisters—to take an occasional Saturday till Monday in bed has the effect of "setting them going again," so that they can face the busy routine of their daily working lives refreshed and rested. It is fortunate when an individual has the good sense to take such a "stitch in time" as this. For neglect of seasonable rest so often results in serious disease, turns an otherwise happy home into a miniature hospital, and may possibly convert all the members of the family into tired and weary nurses.

The nurse electing to undertake the care of rest-cases must be prepared for conditions most trying to health and temper, as the "nervous" rest patient—to put it in the most amiable manner—is perhaps the most selfish and egotistical "human" yet evolved on this earth of marvellous variety of types. For the illness which by a curious paradox has not uncommonly been caused by extreme devotion to duty—by "pegging away" at daily duties incessantly and without due regard to health—seems to bring in its train a complete self-absorption and absolute disregard of others. The unselfishness and self-neglect which have frequently preceded such breakdown have been happily described as "the sins of pious people"—that sin whereby the health is wrecked for lack of a little timely rest and proper wholesome preservation. So that the reaction towards self is often most strongly marked, and patients get into a condition of egotism which amounts almost to a mania. Most rest-cure persons have "I" on the brain, to such a degree that on the one point of self they may almost be regarded as insane. And not only must the nurse do all in her power to take the attention of her patient from the all-absorbing interest he centres in himself and his ever-changing symptoms, but she must, at the same time, show her patient how to control and discipline himself out of this terrible egotism which is so marked a feature of nervous exhaustion.

It is a curious fact—but one which can readily be demonstrated—that one of the first symptoms observable in persons verging towards nervous exhaustion is a persistence in thinking and talking about themselves. And this exaggerated idea of the importance to the world of what they say and think is a never-absent symptom of the nervously prostrated. The advanced form of this "neurotic egotism" is seen in the hysterical and hypochondriacal, and its completed form appears in the insane, who usually take every opportunity of self-aggrandisement, from the belief that they are kings without their lawful crowns, down to the remarkable hallucination that their heads are made of rare and costly porcelain, and are thus more remarkable than those of their neighbours. I once heard a clever nurse remark that "work and duty in a rest-ward suggested a nursing kindergarten. For," said she, "we are constantly giving our patients elementary instruction in the first principles of life. We have to teach them not to weep when they are forbidden food—luxuries after which they hanker, we show them the folly of crying for the moon, and try to lead the hysterical woman—gently but firmly—from her bad habit of screaming for what she wants. So that we are not only nurses—we are elementary teachers in a school for fractious and childish grown-ups."

And what a marvellous amount of patience is needed for such cases. For one has to keep one's serenity during incessant complaints, to amiably answer eternal questions, and to soothe down the frets and friction of a restless, worrying mind which knows no quiet and has lost all power of self-control.

### Educational Congress at the Victorian Era Exhibition.

The subject of the second day's session of the Educational Congress was one of special interest, being the consensus of opinion of ladies who have attained prominence in the medical profession. The chair was taken by Mrs. Fawcett, who fulfilled the duties appertaining to it with quiet dignity.

The Dean of the Royal Free Hospital School of Medicine for Women, Dr. Garrett Anderson, read the first paper, which gave much valuable information to intending lady students. She laid great stress on the necessity for devoting one's whole time during the five years' course to study. There was to be no home nor social distraction, nothing to interfere with regular early hours, wholesome food, and healthy régime.

After the study time was over the newly-fledged doctor would find that she must assimilate the accumulated knowledge, she would have to acquire the art of managing people, and broaden her mind by a wider culture. On the previous day the speaker had had a conversation with the medical superintendent of the Park Hospital at Hither Green, and he had expressed himself as most desirous of having a certain proportion of women doctors on his staff; and it was by such opportunities that the student gained the necessary experience. The financial aspect was one that required careful consideration, as it was not desirable to undertake any other work in order to make a little money.

Mrs. Boyd then read a short address. She was in favour of students having a holiday between the high school cramming and the medical study; she considered a certain amount of social kudos as valuable to the practitioner as essential medical knowledge. Dr. Garrett Anderson differed with her on the ground that the student has a greater facility for passing examinations before twenty-eight than after that age, and because literary and social culture can be attained on the completion of the medical education as before it. Mrs. Boyd, in the course of her address, made a very pertinent remark as to the inadvisability of girls residing at home during their years of study. A conscientious girl is constantly distracted between her duties to her mother and home and her duty towards her work. If at home her refusal to participate in family amusements or employments is regarded as unkind.

Both ladies consider a small sum necessary to set up the student in practice after she obtains her diploma.

Mrs. Scharlieb's paper on "Medical Women in India" was most exhaustive, and we have only space to comment on the most noticeable points. In 1886 there were four medical ladies, herself one of the four, working in India. They were so widely separated that they never met, either for consultation, for advice, or for professional or social converse. Now there are two hundred ladies, native and European, engaged in the healing art. She weighed in an even balance the merits of the Englishwoman and her native sister. As regards brains and keenness for study they were equal; the Englishwoman brought greater self-control, pluck, and a healthy constitution, mentally and morally; but she was handicapped by her ignorance of the people, their language, their environment, the types of disease prevalent, and the climate. The native ladies are gentle and adaptable, but lacking the self-control, courage, and organising power of the Englishwoman, yet they possessed the advantage of local sympathies and knowledge of her people and their modes of life and language. A blending of the two types was desirable, courtesy and patience were especially necessary, for the natives are like children and keen to detect the superficial. Thus it was of the first importance that the women sent to India should be of high and holy characters. It was worthy of note that Indian princes when they founded hospitals in their states invariably asked that they should be officered by native ladies. It was possible that when the daughters of the hardier hill tribes became students, the right sort of medical woman for India would be evolved. Mrs. Scharlieb then spoke pointedly of our duty towards the women and children of India, and bade her hearers remember that in numerous small donations lay the hope of our chronically starved charities. With the inheritance of India's old rulers we were responsible for their duties, and to shut our ears to the cry of India's women and children was nothing less than a national sin. The sick must be healed, the hopeless comforted, and we ought individually to give a proportion of our income to do it.

Unfortunately, medical women might be likened to ironclads; they were a costly production both as regards time and money. An English lady doctor cost five times as much as a native lady doctor, and the demand in time would regulate the supply of both. She was of opinion that English lady doctors should practise in couples, a junior under a senior, as the Indian practitioner has to rely on herself both for skill and nursing, and instead of requiring less knowledge than in England she must be more accomplished. She recommended a long holiday after the completion of the period of study before taking up work.

There was no discussion.

Miss Ellaby's paper was lucid and learned, and full of practical hints useful to all who have the care of young children. It was interesting to hear that the normal standard of sight was practically the same four thousand years ago, and that the faculty of discerning colour is a relatively recent acquisition. These and statistics occupied the first portion of her paper. The second was devoted to the explanation of the organ of sight, of the cause of long and shortsightedness, and the importance of early establishing hygienic conditions and using suitable spectacles when any defect was present; and the last portion was a series of practical suggestions relating to the proper amount of light, the position of the source of light, the proportion and angles of reading and writing desks.

Our readers will be glad to know that they may obtain a verbatim report of all the lectures during the Educational Congress, when ready, from the Secretary, 4, Caroline Place, Mecklenburg Square, W.C., price 2s. 6d.

### Novelties for Nurses.

### A NEW CHART HOLDER.

Messrs. J. Wright and Co., of Bristol, have submitted to us one of their new celluloid chart-holders. In appearance it is neat and effective, the black celluloid contrasting well with the nickle clips, and it is intended that the name of the hospital or institution should be stamped on in gold, which will have an excellent effect. The special advantages of this holder are that it can be cleansed antiseptically; that it is practically indestructible; and that the corners are replaced by clips which are much easier to keep clean. It is made in three thicknesses, according to the size and purpose for which it is required; the strongest being suitable for the largest holders. The price is moderate, ranging from one shilling each, or about as much again as those in leatherette or millboard.

### Queen's Day at the National Hospital for the Paralysed and Epileptic.

By a happy thought of the secretary and general director of this hospital the festival in honour of Her Majesty's long and glorious reign was called the "Queen's Day," and for once there was a change in the incorrect title, "Diamond Jubilee."

At half-past three the handsome out-patients' hall was filled with guests, and shortly after H.R.H. Princess May arrived, exquisitely dressed, looking young and girlish and in the best of health. Lady Mary Lygon and the Hon. Derek Keppel were in attendance. Amongst the guests were the Bishop of Marlborough, the Countess of Bantry, the Right Hon. Evelyn Ashley, P.C., Sir William Gowers, F.R.S., Dr. Ferrier, F.R.S., Dr. Buzzard, Lady Russell Reynolds, Sir Henry and Lady Burdett, General and Mrs. McMahon, Colonel Porter, Lord and Lady Amhurst, Lady Crichton Brown, Mr. Burford Rawlings (the organiser of the fête), the Rev. W. R. Greenhill (chaplain), Miss Rachel Tweed (lady superintendent).

Immediately on the Princess' arrival Mr. Walford Davies sang Mr. Rudyard Kipling's new song, "Our Lady of the Snows," now set to music and sung in public for the first time. Then Madame Antoinette Sterling sang "A Life Lesson," and this was followed in quick succession by songs and instrumental music. The programme was arranged by Miss Janotha, who played two pianoforte solos and accompanied several of the songs herself. Mr. Walford Davies sang another of Rudyard Kipling's songs, "Hymn Before Action," which was also for the first time heard in public. Mr. MacInnes composed the music of both, and had caught the martial swing of the words; they were well received. Madame Antoinette Sterling sang "When I'm Big I'll Be a Soldier," and again, after the Duchess of York and her party had left to inspect the wards, "There is Na Luck About the House." She visited the wards later, and sang to the patients.

Then everyone wandered about the wards, gardens, and corridors as they wished without let or hindrance. Tea, coffee, cakes, and music were provided in the garden and in the board room. Messrs. T. S. Ware, Messrs. Wm. Cutbush, and Messrs. Wm. Paul and Son had sent floral decorations for the wards. The halls, corridors, and "Lady Harriet Bentinck" ward were beautifully adorned with a show of begonias, ferns, and other flowers; the board room and "Princess Christian" ward with Royal roses; the children's ward ("Duchess of Albany") was fragrant with sweet peas. The nurses had worked very hard arranging the flowers, and great taste was shown throughout the hospital. Although the scheme of decorating each ward with one species of flower was good in theory and effective in such wards as had handsome blooms assigned to it, yet some nurses had to use considerable skill to produce a good result where this was not the case. For instance, the "Elizabeth Morgan" ward, which was decorated with cornflowers, and the "Chandler," which had Iceland poppies, owed nearly all their festive appearance to the skill with which the flowers were arranged, for not only, as in the case of the former, the blue was too sombre for effective treatment in this way, but the blooms themselves might have been plucked growing wild, they were so small, whilst the brilliantly-coloured Iceland poppies were too perishable for the purpose. The nurses who arranged them in both cases spent hours before they could obtain any satisfactory result. The gaillardias were fine, and the carnations were fragrant.

In the evening another musical conversazione was arranged by Mr. Allanson Winn, and the gardens were illuminated, but the wards were rightly closed to visitors at a quarter to nine.

Both the parent hospital and the daughter offshoot at East Finchley are free from debt, but efforts are being made to raise £10,000 for permanent improvements and additions, which are much needed.

This class of sickness is one of the most hopeless with which we have to deal, in most cases only a slight improvement can be hoped for, and in others to help the sufferers to bear their lot until a merciful death releases them is all that can be done. The saddest of all perhaps is that the victims are of all ages, and fine handsome children are amongst the babies born to misery.

### Royal British Nurses' Association.

At the quarterly meeting of the General Council held on Friday, the 9th inst., at the offices of the corporation (Sir James Crichton Browne in the chair), the Hon. Medical Secretary (Mr. E. A. Fardon) said his attention had been drawn to certain statements which had recently been published in different papers concerning the Association. One of these emanated from the Central Council of the Incorporated Medical Practitioners' Association, and was signed by the president and the secretary, and the other from certain nurse members of the Association. As legal proceedings were pending against the Editor of the journal of the Association, during the course of which probably some of the points raised in these documents would have to be discussed, it would be improper for him to reply in anything like detail to the charges made. It was not that he was unwilling to reply, but the members of the Association would easily see the position in which he was placed. He would therefore leave those able to form an opinion as to the facts to judge the origin, the accuracy, and the justice of many of the statements contained in these documents. Although he was thus restricted in his reply, he was not restricted from saying that he hoped that every member of the corporation who entertained the opinions expressed in those documents would attend the annual meeting to be held at the Imperial Institute on the 22nd inst., when it would not be his fault if they had no opportunity of expressing their opinions and voting upon them. He and his colleagues, and every member of the Executive Committee, wished that an opportunity should be given at that meeting for the expression of those opinions, and voting on them. He would also say that if they succeeded in carrying their vote of censure against the officials they would have no reason to complain of the action taken by the officials on that occasion.

Dr. Alderson, commenting upon the published statement from the Central Council of the Incorporated Medical Practitioners, said: "I know nothing whatever about it. I wrote to one or two of the prominent members at the council meeting, and they knew nothing about it. I was there yesterday, and I heard that there was nothing in the minutes authorising an important document like this."

After discussions on certain portions of the Executive Committee's and the hon. treasurer's reports, these reports were adopted, and the proceedings shortly afterwards terminated.

### "THE HOSPITAL" CONVALESCENT FUND.

"One who has received benefit from the Fund" sends us a contribution to it of £1, with the cheering news that she is completely restored to health and happy in her work. We rejoice to hear it, and hope that she may continue so.—Nurse E. R. Turner is thanked for her kind contribution of 3s. towards Nurses' Bed. Her good wishes are much appreciated.—We beg to thank Mr. George Lees for his contribution of 1s. towards the above fund. We shall not be able to acknowledge receipt otherwise than in this way, as he has not sent his address.

### Men as Nurses.

By E. Muirhead Little, F.R.C.S.

It is a good many years now since "Martin Chuzzlewit" first furnished an illustration of the evils attendant on a female nurse being placed in charge of a male patient, and of the sufferings of that unfortunate individual in consequence. Old women who drink spirits and prefer to know nothing of their profession have for some time been superseded by young ones, who choose tea, or at the worst wine, as a beverage, and who are apt occasionally to be misled by the amount of valuable information they wish to impart. Lady Priestley's view* of the nursing question, no doubt to some extent inspired by an article in the *St. James's Gazette*, deals with quite another class of dangers from those caused by Mrs. Prig's ignorance of the intricacies of masculine attire and the simplicity of Mrs. Gamp's method of administering a draught. Though the present ills he has to bear may not be so inimical to the sufferer's recovery, yet they expose him to others that he knew not in days gone by. The only surprising point in Lady Priestley's article is that she should call attention to the evil and its remedy as in any sense unforeseen and unprovided for. She dwells at some length and with some insistence on the adoption of nursing as a profession by many women in the middle and lower middle classes as a short and direct road to matrimony; and, unfortunately, many examples can readily be cited to prove the truth of this. [This is a mistaken notion, only a small percentage of nurses marry.—Ed.] But the ennoblement of nursing as a calling began by Miss Nightingale in the early fifties, and developed by her successors into an art, was not unwatched or uncommented upon by male observers. These were chiefly represented by military medical authorities, partly from the fact of their having to rely for army nursing on untrained women, only qualified for their posts by enthusiasm—always more useful as a spur than as a steed—and partly from the necessities of active service which threw the work on men unqualified by anything but a habit of obedience and by goodwill to their fellows.

In 1885 a benevolent lady, touched by the exigencies of the case, founded a philanthropic institution for supplying men with male nurses, and since then associations with a similar object have been springing up in increasing numbers before the growing demand for trained men.

About ten years ago Surgeon-General C. A. Gordon, C.B., now Sir Charles A. Gordon, K.C.B., an officer of nearly forty years' service in the British Army, with a most eventful life in Paris during its siege by the Germans and afterwards during the reign of the Communes, read a paper on the present subject at the Dublin meeting of the British Medical Association. This distinguished officer, as the result of his long and varied experience of paid and of volunteer nurses of both sexes, expressed a decided preference for male attendants, a preference which, he said, was shared by a very large number of sick and wounded men. So much was this the case in Paris that patients would ask to be transferred to a ward where there were no female attendants. In the discussion which followed a remarkable unanimity was shown by the speakers, each bearing testimony to the carefulness, kindness, and skill of the properly trained male nurse.

Although the great novelist's pictures of Mesdames Gamp and Prig were probably as gross caricatures of the nurses of that day as those of Bob Sawyer and Ben Allen were of the medical students and practitioners of the period, yet no one can doubt that intemperance and ignorance were too commonly their characteristics. At the present time the same faults are alleged against male attendants, and the public is consequently prejudiced against them, so that whilst young, pretty, and sometimes frivolous-minded women are unhesitatingly admitted to nurse men, the male nurse is regarded with suspicion. If we inquire into the causes of the great change that fifty years have wrought in the reputation enjoyed by female nurses, we shall find that it is due to systematic training and insistence on the importance and nobility of her calling, which have given to her such a position in the esteem of the public that to-day every woman who wears a nursing dress is assumed as a matter of course to be skilful, sober, and careful. Similar causes will doubtless produce similar results in the case of men, and if proper training can be secured and a sufficient number of well-taught, self-respecting male nurses be forthcoming, the prejudice against them which still lingers will soon die out altogether.

* *Nineteenth Century*, January, 1897.

The metropolitan general hospitals were not slow to appreciate the value of good male nurses and to avail themselves of their services largely in special cases, but they have not so far shown a like alacrity in affording facilities for training men. This difficulty of obtaining proper hospital training for male nurses is one which has confronted those concerned in providing them from the outset, and although it has been to a great extent avoided by engaging men who have been trained in the Medical Staff Corps, and by the kindness of the managers of one large special hospital, it still remains, and limits the number of available trustworthy and thoroughly trained men. It has been suggested, indeed, that a hospital for training purposes such as that of the Deaconesses' Institution at Tottenham might be established, but this scheme was felt to be beyond the scope of any male nursing association besides being open to the objection that it is undesirable to add to the number of small hospitals.

Assuming, then, that female nurses are unfit to nurse many cases of sickness among men, and especially young men, and taking it as proven that men can be taught to be excellent nurses, and that there will be no lack of candidates, the important question remains: Where are they to be properly trained for their duties? That a certain number of well trained men of excellent character are forthcoming from the ranks of the Medical Staff Corps is not a complete answer, for the supply is limited and uncertain, and would entirely cease in case of a great war, when the reservists employed in civil nursing would also be called out. It is at such a time, too, that the services of trained civilians would be most valuable in the voluntary ambulances. Anyone who, like myself, has had the smallest experience in ambulance work in time of war must feel that the weak point of such organisations i in their nursing staff, which, as a rule, has to be improvised from such material as is available on the spot. Female nurses, with all their splendid qualities, of which devotion is not the least, are quite out of place in military hospitals close to or upon the scene of action, and the men who at present form the rank and file of the Volunteer Medical Staff Corps, even though their discipline and theoretical knowledge may be good enough, have with few exceptions no hospital training whatever. Yet hospital training, thanks to the splendid achievements of Lord Lister, is more than ever needed if the work of the surgeon at the operation-table is not to be rendered useless by the nurse's inexperience of the practical details of aseptic and antiseptic surgery. The awful mortality of the best ambulances in the war of 1870-71 is enough to show the importance of strict antiseptic precautions. Nowhere, I believe, are the ravages of blood poisoning in those campaigns more graphically described than in a recent book by Mr. Charles E. Ryan.*

It is obvious that the two sexes cannot be trained together in the same wards, but the problem would be satisfactorily solved if one of the general hospitals would set apart certain wards to be nurs.d by men only and to be used for the instruction of male probationers. If the experiment should prove a success, as I am confident that if properly tried it would be, it could be extended to the whole of the male side of the hospital. If this were done and a male nurses' training school thus established in connection with one of the general hospitals, the intentions of the late Miss Jane Hamilton, of the Hamilton Association, would be carried out, and the society founded and endowed by her might cheerfully hand over its duties and seek a voluntary euthanasia.

Public opinion is beginning to awake at last to the merits of the question. When it is thoroughly aroused it can and will make itself felt, and then the trained male nurse will take that place which he ought to occupy, and in so doing will save the noble calling of the sick nurse from such scandals as those to which Lady Priestley so delicately and appropriately drew attention.

* "With an Ambulance During the Franco-German War." By Charles E. Ryan, F.R.C.S.I. (London: John Murray.)

### The Red Cross and the English Hospitals in Greece.

MANY of the medical men and nurses who went out to the assistance of the Greeks have now returned to England. That they rendered good service goes without saying. In time of war every one who will bear a hand in looking after the wounded is a friend and helper.

However successful war may be its horrors remain, while if unsuccessful these horrors are intensified. Friendly nations may well, then, offer helping hands to the sick and wounded of those engaged in warfare, even when indifferent as to the cause involved. In this case, however, England was not indifferent, and while high politics demanded that we should not actively intervene, the sympathy of England went out to Greece and showed itself in the readiness with which help was sent to the wounded. It was a thing done quickly and done well.

The questions, however, still remain: Was it done as well as it might have been? and is the sending out of complete and independent hospitals the best way of rendering assistance to the wounded in such a case? This is no idle inquiry. He would be a rash man who would assert that the Turko-Grecian difficulty is at an end, and if war should break out again the question would at once arise, What is the best way of rendering service to the wounded? In regard to this it is well to remember that the acceptability of a present depends more on its meeting the wants of the recipient than on the amount it costs, and we do not hesitate to express the opinion that a large amount of the money spent in setting up English hospitals in Greece was thrown away, except so far as the existence of these hospitals was a tangible proof of the sympathy of the English people.

The great mistake was that those responsible for the arrangements did not seem to recognise that, so far as has to do with its town population and so far as the scientific attainments of its professors and the character of the surgical practice in its hospitals are concerned, Greece is a civilised nation. People in England sent out surgeons to do operations, but surgeons were there in plenty; instruments and dressings were also sent out, all of which were already on the spot; and hospitals were set up with separate organisations while hospitals already existed by the score. The Greeks did, however, want nurses, and so far as those who went out fell in with existing organisations, they did well. No one, however, can look back upon events without seeing that more good might have been done had all been under one organisation. People have heard so much of the doings of the little band of English who went to Greece that they are apt to forget how small was the work done in their hospitals in comparison with what was done in the very numerous hospitals established by the Greek ladies working under the Red Cross Society. These ladies were to a large extent untrained, but they were full of patriotism and enthusiasm; they were ladies accustomed to the niceties of life, they had servants at their disposal, and although it is true that the wards of the military hospitals which were nursed by orderlies were dirty to a degree, the Red Cross hospitals which were managed by these ladies were thoroughly well kept, were clean, were properly furnished, and were completely equipped with all surgical dressings and appliances.

So far as the Red Cross hospitals were concerned, and they were both large and numerous, cleanliness and antisepticism seem to have reigned throughout, and even at the military hospitals, although the wards were dirty, all operations seem to have been performed and all dressings done much as they would be at any great civil hospital. The number of surgeons engaged was large, and a considerable number of students also were attached, who performed many of the duties commonly undertaken by nurses in England, such as the preparation of the dressings. In fact, in the military hospitals everything connected with the operations was done by people trained in antiseptic work. There were comparatively few amputations in the Red Cross hospitals, the majority of the operations being for the removal of bullets or for the opening up of compound fractures for the removal of splintered bone. In these cases the skin was prepared by washing with soap followed by spirit of wine or ether, and corrosive sublimate; the wounds were irrigated with corrosive sublimate solution; no sponges were used, nothing but swabs wrung out of sublimate; wounds leading down to bone were generally filled with iodoform gauze, and a dry dressing of the same material was applied and covered with a large wad of cotton wool. All instruments were boiled, and at the time of use were laid in trays of microcidin solution. Notwithstanding that many of the cases had to be removed from Volo to Athens and other places, soon after operation, they did very well, the asepticism being well maintained and very few requiring daily dressing.

There never was any lack of chloroform or other necessaries at Volo in the Red Cross hospital, and stores were freely transferred to the military hospitals when required. We enter into these details to show what we think ought to be recognised, that what the Greeks wanted was not doctors, not hospitals, not stores—all these things they had in abundance. But they did want well-trained nurses to help and guide the great mass of untrained ladies who were giving their services in attending on the sick and wounded, and also to help in the military hospitals. If the necessity should unfortunately arise again, it would be well that any English nurses who go out should work as part of the Red Cross, and not set up separate hospitals outside the official organisation. The wastefulness of arranging a little hospital of twenty-five beds at Piræus was apparent to everyone. The official hospitals in that town and in Athens were in fact so numerous and so large that they could have swallowed up this little affair and all its patients without feeling it. It was not wanted, in fact, but the nurses employed there were very much wanted in other hospitals. Duplicate organisations are always wasteful.

### Everybody's Opinion.

[Correspondence on all subjects is invited, but we cannot in any way be responsible for the opinions expressed by our correspondents. No communication can be entertained if the name and address of the correspondent is not given, or unless one side of the paper only is written on.]

### ROYAL BRITISH NURSES' ASSOCIATION.

The Hon. Medical Secretary, Mr. Fardon, writes: May I ask you to be so kind as to draw your readers' attention to the annual meeting of the Royal British Nurses' Association, which will be held at the East Conference Hall at the Imperial Institute on the 22nd inst. at 11 a.m., when an opportunity will be afforded for discussing the extraordinary statements that have appeared in several papers respecting the management of the association. It is hoped that as many members as possible will attend.

### FEVER NURSING.

"A NORTHERN MATRON" writes: I am very glad to see the paragraph in the last edition of THE HOSPITAL on the necessity of well-trained fever nursing. I am much surprised to find that the large and beautiful hospitals belonging to the M.A.B. in London are not used as training schools, nor do they attempt to keep their nurses. After twelve years' experience of various hospitals, four of which have been spent as matron of a large fever hospital, I am more and more impressed with the valuable training fever nurses have, and also how very little fit a general trained nurse is to take charge of fever cases without first having her fever training. We take nurses for fever training after their general experience, and they constantly say how much they find there is to learn. Our medical men are finding out how very necessary it is for private nurses to have fever experience. I hope before long our large infirmary which grants its nurses certificates without their having seen either enteric or diphtheria cases will include at least six months' fever experience in its three years' curriculum. It is not so very long since utterly untrained women were sent to nurse the fever cases in our hospital because they were not afraid of typhus; now we can select a candidate from among many applicants of a good class, and when our nurses pass on for general work we often hear the remark that "there is more real nursing in our fever hospitals than in the general wards." Our cases are all acute. I hope some other matrons will express their opinions on this subject.

### ALMSHOUSES FOR NURSES.

NURSE BOULTON writes: I think if the public knew how hard was the case of old trained nurses like myself they would give us their help and sympathy. Many of us between sixty and seventy years of age are still most anxious to work for our living, yet time goes on and our cases get fewer and fewer, and we cannot get fresh ones, not because we feel too old for work, but because we are considered to be so. It is natural, I know, that younger faces and younger voices should be more attractive to patients, yet it is very hard that after years of hard work our chance of earning our living should become less, and we have before us the miserable anticipation of having nowhere to lay our heads when our means of income stops altogether. If nurses' almshouses could be instituted, however humble, it would be a great comfort; but it is not for me to suggest what should be done, I simply want to state not only my own case, but the case of those who have worked hard in their profession, and who are getting old, and to whom the prospect of the future is a matter of continual dread.

*** We learn on inquiry that an effort was made to start an almshouse for nurses, assisted through the Junius S. Morgan Benevolent Fund, but was found to be unsatisfactory in result. Practical people do not advocate almshouses. A pension enables the recipient to live where she likes, and does not remove her from her friends. This can be secured by all nurses in the future who cultivate habits of thrift and self-denial when they are young through the Royal National Pension Fund for Nurses, and thus make it unnecessary for them to appeal to charity when working days are over.—ED. *T. H.*

### "BADGES" AGAIN.

"ANOTHER TRAINED NURSE" writes: Before the correspondence on the "Badge" subject is closed, may I say that I do not think there would be very much difficulty in licensing nurses and providing a registered badge, or in meeting the necessary expense. I know that nurses holding London certificates would be only too glad to pay, not only an annual subscription for the privilege of being entitled to wear the badge, but also an entrance fee for the purpose of defraying the initial cost. And most probably our provincial-trained sisters feel the same. The advantages would be great, for, as a nurse could not receive the badge unless she produced her original certificate, the said badge would be proof positive that she was really a trained nurse; and to make assurance doubly sure, let a certificate be given with the badge to prove that it had been given to the nurse displaying it on the production of her certificate of hospital training. No nurse wearing the badge would refuse, if asked, to show her certificate, and unscrupulous people would soon find that it would not pay to imitate the badge. Now if this could be started—say at the office of THE HOSPITAL—with an entrance fee of one guinea and a yearly subscription of the same sum, I do not think there would be many trained nurses who would not gladly apply for the badge. And could there be a better time to begin than the Jubilee year? No trained nurse's salary is too small to bear the expense, and would any of us grudge spending a little less on bicycle riding or whatever happens to be our pet recreation? Those nurses who are not working in London usually take any opportunity to go there, and when in town could apply for the badge; and some of us would go up solely to obtain it. Only a nurse can realise what the lawful possession of such a badge would mean to us. Of course, we should not expect the public to employ exclusively those nurses wearing the badge or to pay them higher fees.

### JUBILEE.

LOUISA STACEY, M.R.B.N.A., writes: I should like those nurses who may be coming to town for the meetings of the Royal British Nurses' Association to know what a boon the Victoria Commemoration Club was to the members on Jubilee Day. Coming from Tunbridge Wells, and arriving at six a.m., I would not have known what to do for two hours and a half but for the club to which I was able to go and wash, brush, rest, and have a comfortable breakfast before starting for my seat. After the procession (which many members who had no seats saw from the club windows) there was a most excellent luncheon provided at moderate charges, and everything was most daintily served and was much appreciated by the crowd of members and their friends in the afternoon, who were able to rest in the cool rooms and dine comfortably before starting to see the illuminations. I cannot help thinking that many nurses will like to join this month and next (who are coming up for meetings or holidays), especially if they hear that the subscriptions will only be half for the remainder of the year and the entrance fee is so small. You know the drawing-room is provided with all kinds of papers, and the cloak-room is a delight to travel-stained nurses, where they find all their wants supplied down to a needle and cotton. I have so far found the club the greatest boon, for, being a private nurse and having no real place of my own, it has been good to belong to a nice club to which I could ask my friends to meet me and to have tea or lunch, as the case may be, and a quiet chat in the pleasant rooms. I am looking forward to the time when bed-rooms will be attached, which, I hear, will be very soon if nurses would only join quickly to make up the necessary number. I am sure I only echo the sentiments of many members when I say we owe it to ourselves (now that someone has so kindly taken the trouble to start such a splendid scheme for us as a profession) to combine and support the pioneer club for nurses. Great praise is due to Miss Fogo Thomson for the way she has worked to make the club what it is in so short a time. She is ever ready to accept suggestions from the members which in any way add to their comfort, and she is doing her utmost to make the club a success, not as a money-making concern, but to pay its way and be a real comfort to nurses.

### Presentations.

A GRACEFUL act of sympathetic appreciation of the endeavours of the nurses at the Stockport Union Infirmary to do well in their work took place on June 25th. The Medical Officer, Mr. W. H. Brady, Dr. W. H. Bale, Mrs. Heys, Mr. H. Brownsword, Mr. Edwards, and a few friends subscribed and bought for Nurses Bishop, Fisher, Macdonald, Jones, and Lewis, who had taken honours in their examinations, handsome chatelaines. The gifts were presented to the nurses at a meeting of the Board of Guardians by the Chairman (Mr. W. H. Brady), and the Matron (Miss Hull) thanked the Guardians on behalf of the recipients.

GRIMSBY.—The committee of the Grimsby and District Nursing Association presented the nurses on both staffs with a souvenir of the Jubilee. They each received a silver pencil case and a silver thimble engraved with their names and "Diamond Jubilee, Grimsby." Nurse Greig, who is leaving, was given, in addition, a gold brooch set with pearls, in recognition of her devoted services to the sick poor of Grimsby.

ESSEX AND COLCHESTER HOSPITAL.—On Monday, 5th inst., at the Essex and Colchester Hospital, Mr. J. E. Bates, house surgeon, was the recipient of a very handsome silver plated preserve and butter stand combined from the matron and the nurses, "a mark of their esteem and regard," on the occasion of his resigning his appointment to enter into private practice.

BLAIRGOWRIE.—Miss Menzies, joint secretary of the local nursing association, was presented with a bicycle for the use of the district nurse, Miss Jackson. The purchase money was collected by Baron Bailie Brown and Mr. T. D. Sharp.

THE committee of the Nurses' Home, 50, Bethel Street, Norwich, commemorated the Diamond Jubilee by giving 10s. and a Thanksgiving Service book to each of their 40 nurses. The kind thought was much appreciated.

### Minor Appointments.

ROYAL ALBERT EDWARD INFIRMARY AND DISPENSARY, WIGAN.—Miss Maud Taylor has been appointed Sister to the children's wards. She was trained at the North-Eastern Children's Hospital, London, for four years. Miss Taylor had, previous to her appointment, been employed in her present capacity since last May, and her conduct and work have been so satisfactory that the appointment was made permanent. For two years Miss Taylor was staff nurse at the hospital where she was trained, and subsequently she joined a private nursing staff, Devonshire Square, London.

SUSSEX COUNTY HOSPITAL, BRIGHTON.—Nurse Elsie Ferrar, has been appointed Sister of the women's medical wards at the Sussex County Hospital, Brighton. She was trained at the City of Cork Hospital for Women and Children and at the Royal Surrey County Hospital, Guildford, where she has held the post of head nurse of the women's wards for the past three years.

WORKHOUSE INFIRMARY, SUNDERLAND.—Miss Anne S. Pruett was appointed Superintendent Nurse on June 24th, 1897.

### Where to Go.

CRANBROOK, KENT.—The Princess Louise, Marchioness of Lorne, and the Marquis of Lorne will open the new Passmore Edwards Convalescent Home of the Metropolitan Hospital at Starvenden Farm, Cranbrook, Kent, on the 20th inst., at half-past four p.m. A special train will leave Charing Cross (S.E.R.) at 2.5 p.m., and will reach Staplehurst at 3.20, where conveyances will be in readiness. The return train will leave Staplehurst at 6.45 p.m., and is timed to reach Charing Cross at 8 p.m.

MIDDLESEX HOSPITAL.—H.R.H. the Princess Christian of Schleswig Holstein will lay the foundation-stone of the new cancer wing at the Middlesex Hospital on Thursday, July 15th, at five p.m. A form of service for the occasion has been drawn up by the Lord Bishop of London.

### Appointments.

MATRONS.

LLOYD COTTAGE HOSPITAL, BRIDLINGTON.—Miss A. Maude Jones was appointed Matron of the above hospital on June 29th. She was trained at Victoria Hospital, Bromley, Lancashire.

### Notes and Queries.

**The contents of the Editor's Letter-box have now reached such unwieldy proportions that it has become necessary to establish a hard and fast rule regarding Answers to Correspondents. In future, all questions requiring replies will continue to be answered in this column without any fee. If an answer is required by letter, a fee of half-a-crown must be enclosed with the note containing the enquiry. We are always pleased to help our numerous correspondents to the fullest extent, and we can trust them to sympathise in the overwhelming amount of writing which makes the new rules a necessity. Every communication must be accompanied by the writer's name and address, otherwise it will receive no attention.**

*Nurses' Uniforms at Netley.*

(316) Will you kindly tell me what uniform is worn indoor and outdoor by the sisters at the Military Hospital, Netley?—*Matthews.*

See answer to A. B., No. 319.

*Lockers.*

(317) Can you tell me of any new lockers that are useful and have a nice appearance? What can you most recommend?—*Matron.*

There are many excellent lockers (see answer to this question in THE HOSPITAL of June 12th, 1897), and a favourite is that made by Messrs. Shoolbred, and used in Poplar Hospital.

*Glasgow Hospitals.*

(318) Would you kindly inform me which hospital in Glasgow would be the best for training in general sick nursing, and the age of entering? (2) Being too young yet, would it be advisable to enter a children's hospital?—*N. Boyd.*

Glasgow has three general hospitals. The Royal Infirmary, with 582 beds, age of admittance, 24; the Victoria Infirmary, with 150 beds, age 24; Western Infirmary, 80 beds, age 24—35. Write to the matrons of each for particulars, and select which suits you best. (2) A year's training at a children's hospital gives experience and prepares a nurse in some degree for work in the wards of a general hospital.

*Army Nurses' Dress.*

(319) (1) Would you kindly tell me the particulars of the Army nurses' dress and (2) if that worn by the Reserve nurses is the same with the addition of the badge? (3) Have any of your correspondents a second-hand edition of "Playfair's Midwifery" and "Bristowe's" to dispose of?—*A. B.*

(1) The indoor uniform of the Army sisters is a grey beige gown, white bibbed apron, a scarlet cape, and a white cap tied under the chin. The outdoor uniform consists of circular cloak, small bonnet, and long veil of grey. The sisters are known as "the Grey Sisters." (2) Each nurse in the Army Nursing Reserve Corps wears her own uniform; the badge is the only difference. (3) This is more a question for advertisement, but we state it in case any of our readers would care to reply.

*Training for Male Nurses.*

(320) Should deem it a kindness if you could tell me where non-paying male probationers are trained as nurses.—*E. S. V. O.*

There is no general training school for male nurses. A limited number of men are trained at the National Hospital for Epilepsy and Paralysis, Queen's Square, W.C. *E. S. V. O.* might also write to Hamilton Association for Providing Trained Male Nurses, 57, Park Street, Grosvenor Square, W., as he might learn the best means of acquiring the training he wants from the secretary.

*Midwifery Training.*

(321) Will you be good enough to inform me the best place to train for monthly nursing; (2) and if there is a chance of earning a living at same?—*E. A. Sorrell.*

(1) The General Hospital, York Road, S.E., bears an excellent reputation, and after the course of monthly nursing is completed, offer the L.O.S. course at half fees. (2) A capable midwife with tact and kindly feeling has every prospect of success.

*District Midwifery.*

(322) I venture to ask your help. I have been trying to work up district midwifery with a view to superseding untrained women. During the first year I had 45 cases, and the second about the same. The committee do not think that the course is being taken advantage of enough to make it worth while keeping me on. Can you offer any suggestion as to how it might be made more popular? Or by inserting query in "Nursing Mirror" find out how similar work is successfully carried on in other places?—*Obstetric.*

Will any of our readers kindly give "Obstetric" their experience?—*Ed. H.*

### Wants and Workers.

NURSE C. T. S., Ewell District Nurse, will be very grateful for any old linen for the use of her patients. The Cave, Ewell, Surrey.

